# Sex-specific association of low-renin hypertension with metabolic and musculoskeletal health in Korean older adults

**DOI:** 10.3389/fpubh.2024.1250945

**Published:** 2024-02-12

**Authors:** Seunghyun Lee, Jae Seung Chang, Kyu-Sang Park, Sang-Baek Koh, Moon Young Kim, Jung Soo Lim

**Affiliations:** ^1^Department of Internal Medicine, Division of Endocrinology and Metabolism, Yonsei University Wonju College of Medicine, Wonju, Republic of Korea; ^2^Department of Sports Science, Hannam University, Daejeon, Republic of Korea; ^3^Department of Physiology, Yonsei University Wonju College of Medicine, Wonju, Republic of Korea; ^4^Department of Preventive Medicine, Yonsei University Wonju College of Medicine, Wonju, Republic of Korea; ^5^Division of Gastroenterology and Hepatology, Department of Internal Medicine, Yonsei University Wonju College of Medicine, Wonju, Republic of Korea; ^6^Regeneration Medicine Research Center, Yonsei University Wonju College of Medicine, Wonju, Republic of Korea; ^7^Cell Therapy and Tissue Engineering Center, Yonsei University Wonju College of Medicine, Wonju, Republic of Korea

**Keywords:** low-renin hypertension, plasma renin activity, primary aldosteronism, bone mineral density, trabecular bone score, sex difference

## Abstract

**Introduction:**

Low-renin hypertension (LRH) accounts for approximately one-third of patients with hypertension and are more prevalent in women and the older adult population. Previous study has found a link between the renin-angiotensin-aldosterone system (RAAS) and sex hormones. However, there are insufficient data on the relationship between LRH and metabolic or musculoskeletal outcomes in older adults.

**Methods and materials:**

Among the 343 participants from a population-based cohort study conducted between May 2018 and August 2019, a total of 256 (86 men older than 50 years and 170 postmenopausal women) were included. The presence of LRH was defined as plasma renin activity (PRA) <1 ng/mL/h and systolic blood pressure (BP) ≥130 or diastolic BP ≥80 mmHg based on the 2017 ACC/AHA guidelines. Individuals with missing data, and those who had used medications that could affect PRA within the past six months were excluded. Bone mineral density (BMD), trabecular bone score (TBS), and appendicular lean mass (ALM) index were assessed using dual-energy X-ray absorptiometry; degraded TBS was defined as partially degraded to degraded levels (≤1.350). Muscle function was assessed according to the Asian Working Group for Sarcopenia guidelines. PRA was measured using radioimmunoassay.

**Results:**

The median age was 66 [61–72] years, and the body mass index (BMI) was 24.7 [23.0–26.4] kg/m^2^. Individuals with LRH, accounting for 34.8%, had lower diabetes mellitus; more dyslipidemia; and poorer muscle function, BMD, and TBS than those in the non-LRH group. In addition, PRA was positively correlated with C-peptide, HOMA-IR, TBS, and ALM index. After adjusting for covariates including age and BMI, LRH was negatively associated with femur neck T-score (adjusted β = −0.30, 95% CI [−0.55 to −0.05], *p* = 0.021) and the presence of LRH was significantly associated with degraded TBS in women (adjusted odds ratio = 3.00, 95% CI [1.36–6.58], *p* = 0.006).

**Conclusion:**

Our findings suggest that LRH can influence clinical features and metabolic risk in older adults. Notably, LRH in postmenopausal women was linked to lower femur neck T-scores and degraded TBS, indicating sex-specific effects of LRH on bone health. Larger prospective studies are required to elucidate how changes in the RAAS affect metabolic and musculoskeletal outcomes in older adults.

## 1 Introduction

The renin-angiotensin-aldosterone system (RAAS) is a circulatory system that is crucial for regulating fluid balance as well as sodium and potassium homeostasis, and its dysregulation may lead to arterial hypertension (1). Classification based on renin levels can help differentiate the pathophysiological causes of hypertension (2). Approximately one-third of patients with hypertension have essential hypertension and low plasma renin activity (PRA), also known as low-renin hypertension (LRH) (3). LRH is more common in women, older adults, and individuals of African descent (4, 5). LRH is a multifactorial disease category that includes patients with a high-sodium diet, primary aldosteronism (PA), hypercortisolism, congenital adrenal hyperplasia, Gordon syndrome, Liddle syndrome, and medications, such as nonsteroidal anti-inflammatory drugs and cyclooxygenase-2 inhibitors (6). Although the final mechanism for LRH development is the activation of mineralocorticoid receptors, which is also linked to the pathogenesis of PA (7), the clinical significance of LRH remains unclear.

The relationship between the RAAS and sex hormones has been widely investigated. Estradiol increases angiotensinogen, angiotensin-converting enzyme 2 (ACE2), and angiotensin type 2 (AT2) receptor expression, while decreasing renin, ACE, and AT1 receptor expression (8, 9). It also acts as an immunomodulator of RAAS (10). Estradiol deficiency after menopause induces low-grade inflammation, which may contribute to the proinflammatory activity of the RAAS and increase oxidative stress (11). Little is known about the effects of androgens on RAAS; however, studies have demonstrated that testosterone activates renin, ACE, and AT1 receptors and inhibits AT2 receptors (12).

Several studies have reported an association among impaired metabolism, musculoskeletal health, and RAAS (13–15). Activation of RAAS may play a central role in the development of obesity; elevated angiotensin II levels are also associated with hypertension, dyslipidemia, and insulin resistance (16). Moreover, bone tissue expresses receptors for RAAS components, such as AT1, AT2, or mineralocorticoid, suggesting that activating the local RAAS response could increase bone turnover, and consequently, bone density (17). Accordingly, LRH is likely to have different clinical features depending on the sex. However, whether LRH is associated with metabolic and musculoskeletal health, particularly among older adults, remains unclear.

Therefore, this study aimed to investigate (1) the differences in clinical features depending on the presence of LRH, (2) the association between RAAS components and metabolic and musculoskeletal parameters, and (3) the sex-specific association of LRH with metabolic and musculoskeletal health in the older adults.

## 2 Materials and methods

### 2.1 Study participants

**Figure 1:**
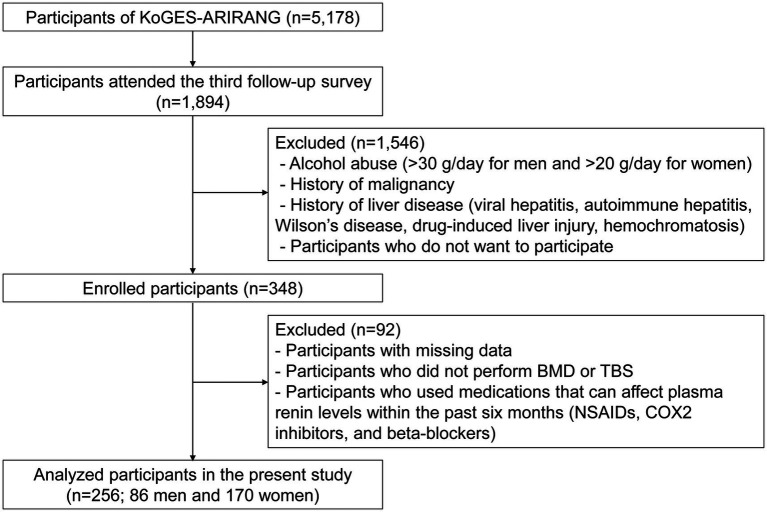
Study flow. KoGES-ARIRANG, the Korean Genome and Epidemiology Study on Atherosclerosis Risk of Rural Areas in the Korean General Population; BMD, bone mineral density; TBS, trabecular bone score; NSAIDs, nonsteroidal anti-inflammatory drugs; COX-2 inhibitors, cyclooxygenase-2 inhibitors.

Study participants were recruited from 1,894 individuals who completed the third follow-up survey (from May 2011 to October 2017) among those already enrolled in the Korean Genome and Epidemiology Study on Atherosclerosis Risk of Rural Areas in the Korean General Population (KoGES-ARIRANG), a population-based cohort study to assess the prevalence, incidence, and risk factors of chronic disorders such as hypertension, diabetes, metabolic syndrome, and cardiovascular disease (18, 19). As shown in Figure 1, among the 348 participants enrolled in the fatty liver cohort, those with missing data, including bone mineral density (BMD) or trabecular bone score (TBS), and those who used medications affecting plasma renin levels within the past six months (nonsteroidal anti-inflammatory drugs and beta-blockers) were excluded. A total of 256 participants (86 men and 170 women) were included for analyses. All included participants were men aged >50 years or post-menopausal women. This study was approved by the Institutional Review Board (IRB), and all study participants provided written informed consent before participation (IRB numbers CR317131, CR318003, and CR322353).

### 2.2 Measurements

Venous blood samples were drawn from the study participants after fasting for >12 h or overnight. Serum aliquots were stored at −80°C freezer until thawed for analysis within one week after blood extraction. A calibrated Roche Cobas^®^ 8,000 modular analyzer consisting of c702a and e801 modules was used to perform routine biochemical tests using the manufacturer’s reagents and calibrators (Roche, Mannheim, Germany). Enzymatic colorimetric techniques were used to assess the triglycerides, total cholesterol, and high-density lipoprotein (HDL) cholesterol levels. Colorimetric techniques were used to measure the aspartate aminotransferase (AST), alanine aminotransferase (ALT), alkaline phosphatase (ALP), creatinine, albumin, blood urea nitrogen (BUN), protein, and total bilirubin levels. The hexokinase method was used to measure the fasting blood glucose levels. An electrochemiluminescence immunoassay was used to measure insulin and C-peptide levels. Molybdate UV and 3 NM-BAPTA techniques were used to quantify calcium and phosphorus, respectively. The formula [fasting insulin (U/mL) × fasting glucose (mg/dL)]/405 was used to calculate the homeostatic model assessment index (HOMA-IR). According to the manufacturer’s instructions, the serum concentrations of fibroblast growth factor 21 (FGF21), FGF19, growth differentiation factor 15 (GDF15), adiponectin, leptin, retinol binding protein 4 (RBP4), interleukin 6 (IL6), transforming growth factor beta 1 (TGF-β1), and myostatin were quantified using the human Quantikine ELISA kits (R&D Systems, Minneapolis, MN, United States) and decorin level was measured using the Raybio human DCN ELISA kit (RayBiotech, Norcross, GA, United States). All mean intra-assay and interassay coefficients of variation (CV) were < 10%.

PRA and aldosterone concentrations were measured using radioimmunoassay (RIA) (Immunotech, Czech Republic), and angiotensin II levels were measured in the sitting position using an ELISA kit (Elabscience, Houston, Texas, United States). The intraassay CV for PRA, aldosterone, and angiotensin II were 11.3, 11.9, and < 10%, respectively, and the inter-assay CVs were 20.9, 10.2%, and < 10%, respectively.

The presence of LRH was defined as a PRA < 1 ng/mL/h and the presence of hypertension, which was defined systolic blood pressure (BP) ≥ 130, or diastolic BP ≥ 80 mmHg based on the 2017 ACC/AHA guidelines or taking antihypertensive medication (20). The non-LRH group included participants with normal-to-high renin (PRA ≥ 1 ng/mL/h) hypertension as well as those without hypertension. BMD and TBS were measured using dual-energy X-ray absorptiometry (DXA); degraded TBS was defined as partially degraded to degraded levels (≤1.350) (21). The Short Physical Performance Battery (SPPB), usual gait speed, timed-up-and-go test, 5-timed chair stand test were assessed according to the Asian Working Group for Sarcopenia guidelines (22).

### 2.3 Statistical analyses

Data are presented as mean ± standard deviation (SD), median [interquartile range (IQR)], or numbers (percentages) in Table 1 and Supplementary Table S1. Independent *t*-test, Wilcoxon rank-sum test, or Pearson’s chi-square test were performed, as appropriate, to compare the clinical characteristics between the LRH and non-LRH groups. The correlations between the RAAS components and metabolic and musculoskeletal parameters were assessed using the Spearman correlation coefficient (r). Univariate and multivariate regression models were used to investigate the association between the presence of LRH and femur neck T-scores as well as muscle function tests. Univariate and multiple linear regression analyses were performed to determine whether the presence of LRH influenced the degraded TBS. We used STATA 17.0 (Stata Corp LP, College Station, TX, United States) in all analyses. All statistical tests were two-sided, and the significance level was set at *p* < 0.05.

**Table 1:** Baseline characteristics.

	LRH (*n* = 89)	Non-LRH (*n* = 167)	Total (*n* = 256)	*p*-value
Age (y)	67 (62–72)	65 (61–72)	66 (61–72)	0.284
Sex [men, *n* (%)]	22 (24.7%)	64 (38.3%)	86 (33.6%)	0.028*
HTN med (*n*, %)	52 (58.4%)	67 (40.1%)	119 (46.5%)	0.005*
DM (n, %)	10 (11.2%)	42 (25.1%)	52 (20.3%)	0.008*
Dyslipidemia (*n*, %)	47 (52.8%)	61 (36.5%)	108 (42.2%)	0.012*
Osteoporosis (*n*, %)	24 (27.0%)	30 (18.0%)	54 (21.1%)	0.093
PRA (ng/mL)	0.6 (0.3–0.7)	1.2 (0.6–2.9)	0.8 (0.5–1.6)	<0.001*
Aldosterone (pg/mL)	15.6 (12.8–19.1)	14.8 (11.9–18.9)	15.2 (12.0–19.0)	0.348
Angiotensin II (pg/mL)	66.6 (42.7–115.2)	75.7 (58.3–105.6)	74.4 (53.7–108.2)	0.189
AST (U/L)	22 (20–26)	22 (19–26)	22 (19–26)	0.732
ALT (U/L)	18 (14–22)	18 (13–23)	18 (13–23)	0.806
Cr (mg/dL)	0.7 (0.6–0.8)	0.8 (0.6–0.9)	0.7 (0.6–0.9)	0.386
Metabolic stress-related biomarkers				
RBP4 (μg/mL)	29.7 (26.0–36.2)	31.2 (27.1–35.9)	30.7 (26.8–36.0)	0.450
IL6 (pg/mL)	1.65 (1.19–2.41)	1.44 (1.04–2.31)	1.53 (1.08–2.35)	0.107
Myostatin (pg/mL)	2.70 (2.08–3.16)	2.59 (2.00–3.38)	2.61 (2.03–3.31)	0.965
Tgfb1 (pg/mL)	24.0 (20.5–28.9)	24.3 (19.5–30.0)	24.2 (20.0–29.4)	0.734
Decorin (pg/mL)	6.9 (5.9–8.2)	6.9 (5.9–7.8)	6.9 (5.9–8.0)	0.348
GDF15 (pg/mL)	859 (708–1,027)	849 (656–1,197)	854 (671–1,151)	0.784
FGF19 (pg/mL)	169 (109–289)	172 (110–311)	170 (110–301)	0.889
FGF21 (pg/mL)	235 (133–318)	183 (119–301)	191 (127–317)	0.156
Metabolic parameters				
BMI (kg/m^2^)	24.4 (23.3–26.3)	24.9 (23.0–26.6)	24.7 (23.0–26.4)	0.783
Total fat (%)	38.5 (32.9–41.7)	36.8 (30.3–40.6)	37.3 (30.8–41.3)	0.082
Glucose (mg/dL)	98 (92–105)	100 (93–110)	99 (93–110)	0.200
Triglyceride (mg/dL)	114 (89–167)	129 (90–212)	125 (90–192)	0.196
Total cholesterol (mg/dL)	179 ± 35	176 ± 35	177 ± 35	0.502
HDL cholesterol (mg/dL)	54 (48–61)	51 (42–62)	52 (42–62)	0.158
C-peptide (ng/mL)	1.9 (1.6–2.8)	2.2 (1.5–3.2)	2.1 (1.5–3.1)	0.186
Insulin (uIU/mL)	6.8 (4.7–11.6)	7.3 (5.1–12.0)	7.1 (5.1–11.9)	0.363
HOMA-IR	1.6 (1.0–2.9)	1.8 (1.2–3.2)	1.7 (1.2–3.1)	0.203
HOMA-β	74 (52–111)	69 (49–105)	72 (50–107)	0.537
Adiponectin (ng/mL)	7.6 (4.2–11.9)	6.1 (3.5–9.9)	6.4 (3.7–10.8)	0.123
Leptin (ng/mL)	9.6 (4.4–14.1)	7.3 (3.8–12.9)	8.1 (3.9–13.5)	0.072
Musculoskeletal parameters				
Ca (mg/dL)	9.5 ± 0.3	9.6 ± 0.3	9.5 ± 0.3	0.090
P (mg/dL)	3.7 ± 0.4	3.8 ± 0.5	3.8 ± 0.4	0.693
ALP (IU/L)	66 (58–80)	70 (56–81)	68 (57–81)	0.365
ALM index (kg/m^2^)	5.6 (5.1–6.4)	5.9 (5.2–6.7)	5.7 (5.2–6.6)	0.147
Handgrip strength (kg)	23.6 (19.5–29.4)	26.8 (21.8–34.5)	25.7 (20.6–33.4)	0.009*
Gait speed (m/s)	1.04 (0.96–1.16)	1.05 (0.95–1.16)	1.05 (0.95–1.16)	0.967
Chair stand test (sec)	10.7 (8.3–13.6)	9.4 (8.3–11.5)	10.0 (8.3–12.5)	0.031*
TUG time (sec)	8.8 (8.3–10.5)	8.8 (7.9–9.8)	8.8 (8.1–9.9)	0.080
SPPB (score)	11 (11–12)	12 (11–12)	12 (11–12)	0.004*
TBS	1.33 (1.28–1.39)	1.37 (1.31–1.41)	1.36 (1.30–1.41)	0.024*
Lumbar spine T-score	−1.0 (−2.1 to 0.2)	−1.0 (−1.9 to −0.1)	−1.0 (−2.0 to 0.0)	0.868
Femur neck T-score	−1.7 (−2.2 to −0.7)	−1.3 (−1.9 to −0.5)	−1.4 (−2.0 to −0.6)	0.020*
Total hip T-score	−0.7 (−1.3 to −0.1)	−0.5 (−1.1 to 0.3)	−0.7 (−1.1 to 0.2)	0.095
Fracture history (*n*, %)	18 (20.7%)	27 (16.9%)	45 (17.6%)	0.458
Nontraumatic fracture history (*n*, %)	7 (8.1%)	10 (6.2%)	17 (6.6%)	0.585

Comparisons between groups using the Independent two sample t-test, Wilcoxon rank sum test or Pearson’s chi-square tests. * indicates a significant difference *p* < 0.05. LRH, low renin hypertension; y, years; HTN med, antihypertensive medication; DM, diabetes mellitus; PRA, plasma renin activity; AST, aspartate aminotransferase; ALT, alanine aminotransferase; Cr, creatinine; RBP4, retinol binding protein 4; IL6, interleukin-6; Tgfb1, transforming growth factor beta 1, GDF15, growth and differentiation factor 15; FGF19, fibroblast growth factor 19; FGF21, fibroblast growth factor 21; BMI, body mass index; HOMA-IR, Homeostatic Model Assessment for Insulin Resistance; HOMA-β, Homeostatic Model Assessment for beta cell function; Ca, calcium; P, phosphorus; ALP, alkaline phosphatase; ALM index, appendicular lean mass index; TUG time, timed up and go time; SPPB, short physical performance battery; TBS, trabecular bone score.

## 3 Results

### 3.1 Clinical characteristics of the study participants

**Figure 2:**
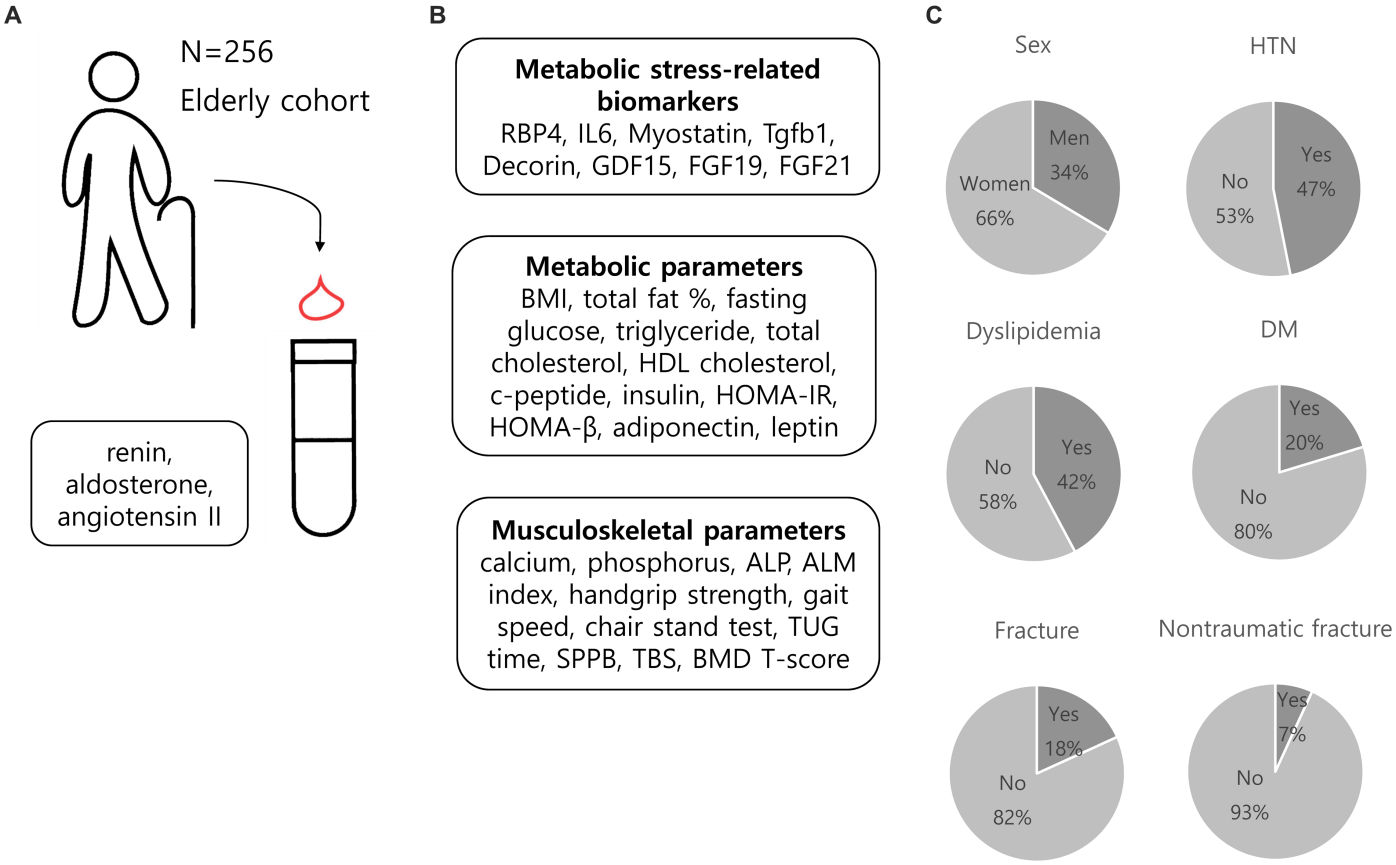
Overview of the cohort of the current study. **(A)** We measured renin, aldosterone, and angiotensin II levels in 256 older adults participants. **(B)** Metabolic stress-related parameters, metabolic parameters, and musculoskeletal parameters were also evaluated. **(C)** Graphical representation of baseline characteristics of the cohort. RBP4, retinol binding protein 4; IL6, interleukin-6; Tgfb1, transforming growth factor beta 1, GDF15, growth and differentiation factor 15; FGF19, fibroblast growth factor 19; FGF21, fibroblast growth factor 21; BMI, body mass index; HDL cholesterol, high-density lipoprotein cholesterol; HOMA-IR, Homeostatic Model Assessment for Insulin Resistance; HOMA-β, Homeostatic Model Assessment for beta cell function; ALP, alkaline phosphatase; ALM index, appendicular lean mass index; TUG time, timed up and go time; SPPB, short physical performance battery; TBS, trabecular bone score; BMD, bone mineral density; HTN, hypertension; DM, diabetes mellitus.

Among the 256 participants, 170 (66.4%) were women, 120 (46.9%) had hypertension, and 89 (34.8%) had LRH (Figure 2). The median age of study population was 66 [61–72] years, and the body mass index (BMI) was 24.7 [23.0–26.4] kg/m^2^. Men accounts for 24.7 and 38.3% in the LRH and non-LRH groups, respectively, and the difference was significant (*p* = 0.028). Individuals with LRH were prescribed more antihypertensive medications, had less diabetes mellitus (DM) and more dyslipidemia than non-LRH group (antihypertensive medication, 58.4% vs. 40.1%, *p* = 0.005; DM, 11.2% vs. 25.2%, *p* = 0.008; dyslipidemia, 52.8% vs. 36.5%, *p* = 0.012) (Table 1). When stratified according to sex, no significant difference was observed in the prevalence of DM in the presence of LRH (Supplementary Table S1). Participants with LRH had poorer handgrip strength, chair stand time, SPPB, TBS, and femur neck T-score than those in the non-LRH group (handgrip strength, 23.6 [19.5–29.4] vs. 26.8 [21.8–34.5], *p* = 0.009; chair stand time, 10.7 [8.3–13.6] vs. 9.4 [8.3–11.6], *p* = 0.031; SPPB, 11 (11–12) vs. 12 (11–12), *p* = 0.004; TBS, 1.33 [1.28–1.39] vs. 1.37 [1.31–1.41], *p* = 0.024; femur neck T-score, −1.7 [−2.2 to −0.7] vs. –1.3 [−1.9 to −0.5], *p* = 0.020). TBS and femur neck T-score were significantly lower in the LRH than in non-LRH group (TBS, 1.33 [1.28–1.39] for LRH group vs. 1.37 [1.31–1.41] for non-LRH group, *p* = 0.024; femur neck T-score, −1.7 [−2.2 to −0.7] for LRH group vs. −1.3 [−1.9 to −0.5] for non-LRH group, *p* = 0.020) (Table 1). Women with LRH had more dyslipidemia and lower TBS and femur neck T-score compared with women without LRH; meanwhile, there were no significant differences in T-score and TBS between men with and without LRH (Supplementary Table S1).

### 3.2 Correlation between components of RAAS and each metabolic stress-related, metabolic, and musculoskeletal parameter in older adults

**Figure 3:**
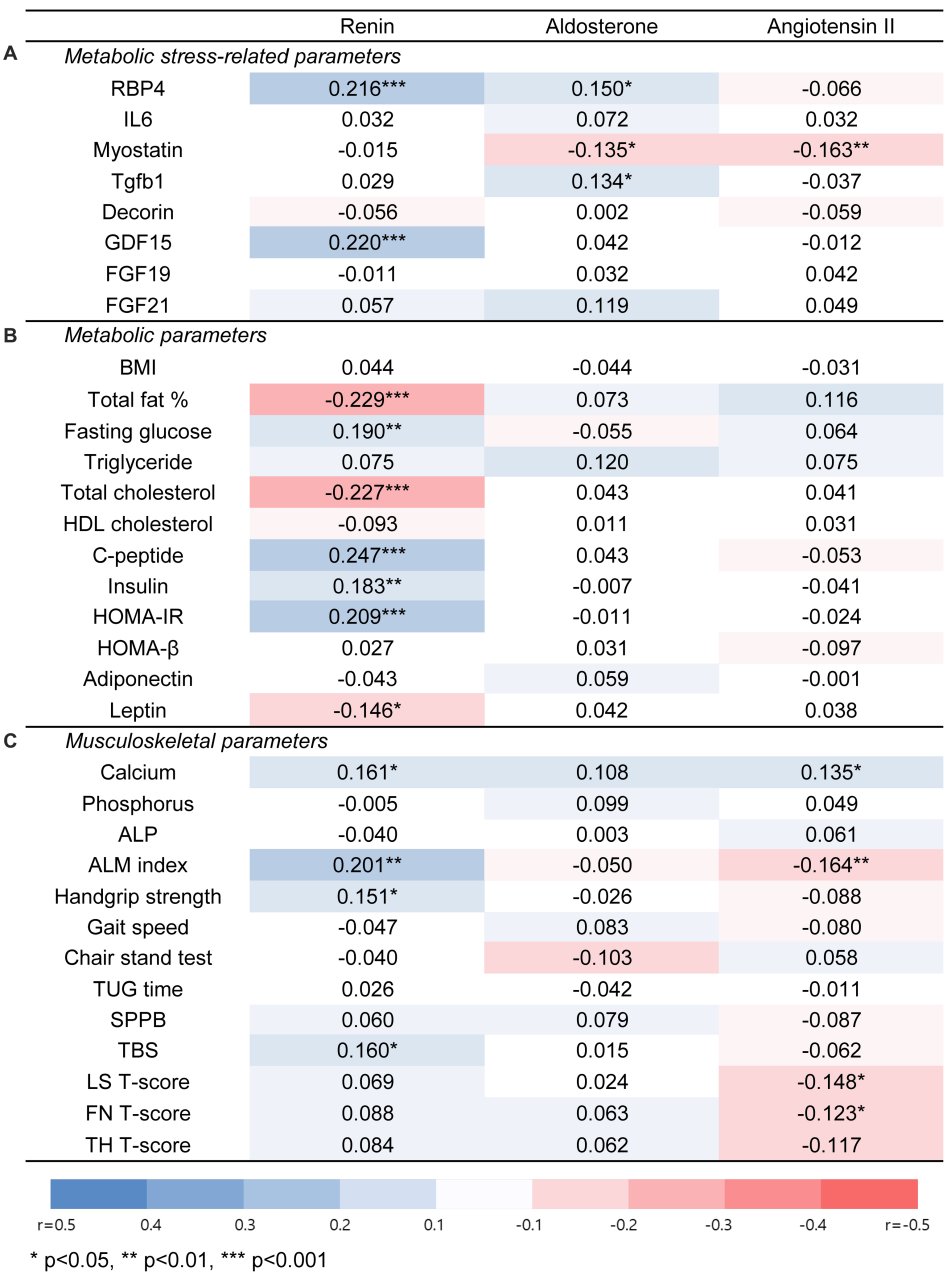
Associations of renin, aldosterone, and angiotensin II with metabolic stress-related parameters, metabolic, or musculoskeletal parameters in total population. **(A)** Metabolic stress-related parameters. **(B)** Metabolic parameters. **(C)** Musculoskeletal parameters. Significant correlations between each parameter were evaluated by spearman correlation analysis. An orange square indicates a positive association, and a blue square indicates a negative association. Abbreviations: RBP4, retinol binding protein 4; IL6, interleukin-6; Tgfb1, transforming growth factor beta 1; GDF15, growth and differentiation factor 15; FGF19, fibroblast growth factor 19; FGF21, fibroblast growth factor 21; BMI, body mass index; HOMA-IR, Homeostatic Model Assessment for Insulin Resistance; HOMA-β, Homeostatic Model Assessment for beta cell function; ALP, alkaline phosphatase; ALM index, appendicular lean mass index; TUG time, timed up and go time; SPPB, short physical performance battery; TBS, trabecular bone score; LS, lumbar spine; FN, femur neck; TH, total hip. * for *p* < 0.05, ** for *p* < 0.01, *** for *p* < 0.001.

The correlation between the RAAS components and each parameter was investigated to determine whether RAAS affects the metabolic and musculoskeletal health of older adults (Figure 3).

PRA was positively associated with glucose, C-peptide, insulin, and HOMA-IR levels, which are parameters related to insulin secretion or resistance, whereas there was a negative correlation between PRA and total fat or total cholesterol. Angiotensin II levels were positively correlated with serum calcium levels and negatively correlated with the appendicular lean mass index (ALM index). In contrast, aldosterone levels showed no significant association with most parameters.

In a multivariable regression analysis adjusted for age, sex, BMI, use of antihypertensive medication, presence of DM or dyslipidemia, and serum creatinine level, the associations that remained significant were between PRA and fasting glucose levels (adjusted β = 1.133 [0.530–1.735], *p* < 0.001), PRA and HOMA-IR (adjusted β = 0.137 [0.040–0.233], *p* = 0.006), as well as angiotensin II and serum calcium level (adjusted β = 0.0003 [0.0001–0.0007], *p* = 0.020).

### 3.3 Association between the presence of LRH and musculoskeletal health among older adults

As shown in Table 2, femur neck T-score showed a significant negatively associated with the presence of LRH in women (unadjusted β = −0.31, 95% CI [−0.57, −0.04], *p* = 0.022). In multiple regression analysis, the femur neck T-score also showed a significant association with LRH, independently of age, BMI, the presence of diabetes or dyslipidemia, the use of antihypertensive medication, and serum creatinine level in women (adjusted β = −0.29, 95% CI [−0.54 to −0.04], *p* = 0.025) (Table 2). However, lumbar spine T-score, total hip T-score, and TBS did not seem to show a significant association with the presence of LRH in univariable regression analysis in women (lumbar spine T-score, unadjusted β = 0.03, 95% CI [−0.03 to 0.40], *p* = 0.884; total hip T-score, unadjusted β = −0.23, 95% CI [−0.50 to 0.04], *p* = 0.091; TBS, unadjusted β = −0.02, 95% CI [−0.04 to 0.00], *p* = 0.064).

**Table 2:** Results of univariable and multivariable regression analysis of femur neck T-score.

(A) Men				
Femur neck T-score	Unadjusted		Adjusted	
	β coefficient (95% CI)	*p*-value	β coefficient (95% CI)	*p*-value
The presence of LRH	0.20 (−0.29 to 0.69)	0.413	0.22 (−0.23 to 0.66)	0.335
Age (years)	−0.04 (−0.07 to −0.01)	0.006*	−0.04 (−0.07 to −0.01)	0.007*
BMI (kg/m^2^)	0.14 (0.07 to 0.21)	<0.001*	0.14 (0.07 to 0.21)	<0.001*
HTN med	−0.09 (−0.52 to 0.34)	0.675	−0.21 (−0.61 to 0.18)	0.289
The presence of DM or dyslipidemia	0.02 (−0.41 to 0.45)	0.912	0.06 (−0.34 to 0.45)	0.772
Cr (mg/dL)	0.00 (−1.32 to 1.33)	0.996	0.02 (−1.16 to 1.21)	0.970

(B) Women				
Femur neck T-score	Unadjusted		Adjusted	
	β coefficient (95% CI)	*p*-value	β coefficient (95% CI)	*p*-value
The presence of LRH	−0.31 (−0.57 to −0.04)	0.022*	−0.29 (−0.54 to −0.04)	0.025*
Age (years)	−0.05 (−0.07 to −0.03)	<0.001*	−0.05 (−0.07 to −0.03)	<0.001*
BMI (kg/m^2^)	0.08 (0.04 to 0.13)	<0.001*	0.07 (0.03 to 0.11)	0.002*
HTN med	0.00 (−0.26 to 0.27)	0.983	0.18 (−0.08 to 0.44)	0.173
The presence of DM or dyslipidemia	−0.10 (−0.47 to 0.28)	0.612	0.04 (−0.21 to 0.29)	0.755
Cr (mg/dL)	−0.39 (−1.58 to 0.81)	0.522	0.08 (−1.02 to 1.17)	0.887

Univariable and multivariable analyses using linear regression model adjusted for age, BMI, the use of hypertension medication, the presence of DM or dyslipidemia, and serum creatinine level. ^*^indicates significant association *p* < 0.05. 95% CI, 95% confidence interval; LRH, low renin hypertension; BMI, body mass index; HTN med, antihypertensive medication; DM, diabetes mellitus; Cr, serum creatinine.

Considering the possibility that a significant association could not be shown due to the small size of TBS, logistic regression analysis was performed by classifying as degraded TBS (≤ 1.350) or non-degraded TBS (> 1.350). Univariate logistic regression analysis revealed a significant correlation between the presence of LRH and a degraded TBS in women. Moreover, even after adjusting for age, BMI, the presence of diabetes or dyslipidemia, the use of antihypertensive medication, and serum creatinine level, the association between the presence of LRH and degraded TBS in women remained significant (adjusted odds ratio = 3.22, 95% CI [1.46–7.11], *p* = 0.004) (Table 3). In men, the presence of LRH did not show any correlation with BMD T-scores or TBS.

**Table 3:** Results of univariable and multivariable logistic regression analysis of degraded TBS (≤1.350).

(A) Men				
Degraded TBS	Unadjusted		Adjusted	
	β coefficient (95% CI)	*p*-value	β coefficient (95% CI)	*p*-value
The presence of LRH	0.17 (0.02–1.38)	0.097	0.17 (0.20–1.40)	0.100
Age (years)	1.05 (0.97–1.14)	0.238	1.05 (0.96–1.15)	0.260
BMI (kg/m^2^)	0.97 (0.80–1.19)	0.797	0.95 (0.77–1.17)	0.635
HTN med	2.30 (0.71–7.42)	0.162	2.35 (0.68–8.10)	0.176
The presence of DM or dyslipidemia	1.05 (0.34–3.21)	0.931	0.78 (0.23–2.61)	0.684
Cr (mg/dL)	1.03 (0.03–32.13)	0.986	0.84 (0.03–23.26)	0.919

(B) Women				
Degraded TBS	Unadjusted		Adjusted	
	β coefficient (95% CI)	*p*-value	β coefficient (95% CI)	*p*-value
The presence of LRH	2.39 (1.21–4.73)	0.012*	3.22 (1.46–7.11)	0.004*
Age (years)	1.07 (1.01–1.12)	0.013*	1.08 (1.02–1.15)	0.007*
BMI (kg/m^2^)	1.01 (0.90–1.14)	0.814	1.05 (0.93–1.20)	0.410
HTN med	0.81 (0.43–1.54)	0.519	0.40 (0.18–0.88)	0.023*
The presence of DM or dyslipidemia	0.64 (0.27–1.56)	0.329	0.94 (0.46–1.92)	0.874
Cr (mg/dL)	2.71 (0.13–55.09)	0.517	1.21 (0.04–36.22)	0.912

Univariable and multivariable analyses using logistic regression model adjusted for age, BMI, the use of hypertension medication, the presence of DM or dyslipidemia, and serum creatinine level. *indicates significant association *p* < 0.05. TBS, trabecular bone score; 95% CI, 95% confidence interval; LRH, low renin hypertension; BMI, body mass index; HTN med, antihypertensive medication; DM, diabetes mellitus; Cr, serum creatinine.

There was no significant relationship between the presence of LRH and muscle function (handgrip strength, chair stand test, and SPPB), as well as muscle mass when analyzed using univariate and multivariate regression analyses.

## 4 Discussion

Our findings suggest that sex differences in metabolic and musculoskeletal health among older adults were found according to the presence of LRH. Unlike in older adults men, LRH was associated with lower femur neck T-score and degraded TBS in postmenopausal women, indicating a higher risk of osteoporosis and future fracture.

LRH encompasses a wide spectrum of disorders, but common causes of LRH encountered in clinical practice include PA, a high-sodium diet, and some medications known to disturb RAAS (6). LRH and PA share a common mechanism of mineralocorticoid activation, which results in a higher risk of cardiovascular disease and mortality (23, 24). PA has an unrecognized prevalence rate of 6%–17% even in normotensive individuals (25). Similar to PA, LRH is associated with a higher incidence of cardiovascular diseases than essential hypertension (23, 24). In addition to PA, several studies have reported an association between high sodium intake and adverse cardiovascular mortality and morbidity (26). This association may be attributed to increased extracellular sodium concentrations, potentially leading to adverse effects on vascular reactivity and growth as well as the stimulation of myocardial fibrosis (27). A decrease in RAAS activity with age is also associated with a reduction in renin levels, leading to a lower PRA (28). Consequently, LRH is more prevalent and significant in older adults (4, 5).

Previous studies on the effects of LRH on metabolic components such as diabetes and dyslipidemia have been conducted; however, this subject remains controversial. A study of 275 adolescents and adults without classic PA reported that PRA was negatively correlated with age, BMI, percent body fat, waist-to-hip ratio, and low-density lipoprotein (LDL) cholesterol (29). The negative correlation between PRA and percent body fat is also consistent with our results. However, according to Monticone et al. (3) there were no significant differences in metabolic parameters, including blood glucose, triglyceride, and LDL cholesterol levels between LRH and normal-high renin groups. This difference may be attributed to the age discrepancy of the participants and the PA inclusion between the two studies. In our study, the positive association between PRA and glucose and the lower prevalence of diabetes in participants with LRH might be explained by previous literature showing that diabetic patients have higher PRA levels than normal controls (30). Furthermore, there was a difference in muscle function between the LRH and non-LRH groups; however, this difference was not statistically significant in the multivariate regression analysis. This could potentially be due to the impact of bone deterioration and the inclusion of individuals with probable PA; osteoporosis is associated with decreased muscle function (31). Muscle weakness can occur in patients with PA, particularly when the plasma concentration of potassium is less than 2.5 meq/L (32).

Previous studies have reported the relationship between LRH and bone health. Tylavsky et al. reported a positive correlation between PRA and distal BMD in premenopausal women with high sodium intake, indicating the negative metabolic effects of LRH on the bone (14). In addition, Kuipers et al. (15) demonstrated a significant association between PRA and BMD or bone turnover markers in the general population, with a positive correlation between PRA and trabecular volumetric BMD measured by quantitative computed tomography, and a negative correlation between PRA and osteocalcin levels in 373 African ancestry family members. However, these two studies included relatively young age groups. In contrast, our research was conducted in an older adult population where LRH is more common but seldom studied and showed sex differences.

Interestingly, in our study, LRH was associated with lower femur neck T-score and degraded TBS in postmenopausal women. TBS is a measure that assesses changes in the gray-scale intensity of pixels in DXA images of the lumbar spine, which indirectly reflects the microarchitecture of the trabecular bone, and it has the potential to enhance fracture prediction compared to using DXA alone (21). The mechanism underlying the association between LRH and bone quantity or quality has not yet been elucidated, but the following three mechanisms are possible. First, it is possible that a large number of PA were included, as mentioned earlier. In our study, probable PA, defined as PRA < 1 ng/mL/h and ARR of 20 or higher, was significantly higher in LRH group than in non-LRH group (70 [78.7%] in the LRH group vs. 48 [28.7%] in the non-LRH group, *p* < 0.001). PA may deteriorate bone health through (1) secondary hyperparathyroidism due to increased urinary calcium (33); (2) a direct effect on bone health through the distribution of mineralocorticoid receptors in human osteoclasts, osteoblasts, osteocytes, and parathyroid tissue (34, 35); and (3) reduced bone formation and increased apoptosis of osteoblasts and osteocytes caused by inflammation due to oxidative stress (36, 37). PA is a well-known risk factor for fractures (38), and a significant number of PA may have influenced the study results. Second, some participants consuming a high-sodium diet may also have affected our findings. High sodium intake can cause LRH by inhibiting renin activity and aldosterone release (39). In addition, increased sodium intake may increase urine calcium excretion, which can accelerate bone remodeling and loss (40). A previous meta-analysis showed a positive association between sodium intake and osteoporosis risk (41). Third, increased local angiotensin II activity in patients with LRH may affect bone deterioration (32). In patients with LRH, the activity of angiotensin II can rise locally within certain tissues, such as the vascular endothelium, kidneys, and adrenal glands, even if angiotensin II levels are normal (32). Although no significant difference in angiotensin II level was observed between the LRH and non-LRH groups, there was a significant association between angiotensin II and lumbar spine T-score, as well as femur neck T-scores in this study. Angiotensin II may influence bone cells by binding to AT1 receptors on osteoblasts and triggering the release of mediators that activate osteoclasts, which are thought to modulate blood flow in the bone marrow capillaries and contribute to osteoclastic bone resorption (42, 43). Moreover, angiotensin II potentially affects calcium metabolism by elevating parathyroid hormone and decreasing ionized calcium levels (44, 45).

The reasons for sex differences in the relationship between LRH and BMD or bone quality may include the following: first, women with relatively large sex hormonal variations might be more vulnerable to the harmful impacts of excessive aldosterone compared to men. Kim et al. demonstrated that only women with PA showed a lower TBS than those with nonfunctioning adenomas (46). Second, RBP4, which is significantly associated with PRA in women, may influence sex differences. In particular, the association between PRA and RBP4 levels may explain why BMD and PRA are associated only in women. RBP4 is secreted by adipocyte, and *in vivo* studies have demonstrated that chondrocytic RBP4 is involved in bone growth (47). However, in clinical studies, the role of RBP4 in bone health appears to vary with age and sex. Some studies reported a positive association between RBP4 and BMD in postmenopausal women with osteopenia or osteoporosis (48, 49). In contrast, another study in young men did not show any association between RBP4 and BMD (50). Furthermore, serum GDF15 level, which may affect bone metabolism or muscle homeostasis in old women via inflammatory responses (51), seemed to have positive linear correlation with the presence of LRH in the current study. Further research is essential to understand the role of RBP4 and GDF15 levels on musculoskeletal health in the older adults with LRH.

To our knowledge, this is the first clinical study to report a significant relationship between PRA and bone quality as well as BMD in older women, but not in older men. Additionally, while sex differences in hypertension and cardiovascular disease due to differential activation of the sympathetic nervous system, RAAS, and immune system have been previously acknowledged (52, 53), this is the first report to indicate a sex difference in the relationship between the presence of LRH and bone health in older participants. However, this study has several limitations. First, it was difficult to infer causality owing to the cross-sectional research design. Second, the sample size was small, which might in part have affected the insignificant results, especially in men, which may limit the generalizability and statistical power of the current results. Therefore, future research with a larger and more diverse samples will be required to enhance the applicability of our findings. Third, although liquid chromatography-mass spectrometry is more accurate and reliable than RIA for the measurement of PRA (54), we measured PRA using RIA because of its cost and availability. Fourth, hypercortisolism may have been included in the low renin-low aldosterone patient group (6), which may have affected the study results. Also, we did not investigate the form of could not precisely exclude secondary hypertension. Fifth, unfortunately, the specific medication profiles for all hypertensive patients were not retrieved, with only partial data available for some individuals. Finally, owing to the lack of data on 25-hydroxyvitamin D, parathyroid hormone levels, serum electrolyte levels, bone turnover markers, calcium/vitamin D supplementation, urinary sodium excretion, or sodium intake, the mechanisms by which LRH adversely affect bone density or quality cannot be well explained. However, an age-related decrease in the RAAS has been found in normal participants, regardless of sodium repletion (55).

In summary, our research suggests that clinical characteristics and metabolic risk in older adults could be influenced by the presence of LRH. LRH in postmenopausal women was associated with a lower femur neck T-score and degraded TBS, highlighting the sex-specific impact of LRH on bone health in old women. A more comprehensive, larger, prospective study is needed to clarify how RAAS affects metabolic and musculoskeletal outcomes in the older adult population.

## Data availability statement

The raw data supporting the conclusions of this article will be made available by the authors, without undue reservation.

## Ethics statement

The studies involving humans were approved by this clinical study was permitted by the Institutional Review Board (IRB) of Yonsei University Wonju College of Medicine (IRB numbers CR317131, CR318003, and CR322353). The studies were conducted in accordance with the local legislation and institutional requirements. The participants provided their written informed consent to participate in this study.

## Author contributions

JC, MK, K-SP, S-BK, and JL conceptualized and designed the study. SL, JC, MK, and JL have conducted the study. SL, JC, MK, K-SP, S-BK, and JL have collected and interpreted data. SL and JL have drafted the manuscript and have revised and finalized the manuscript. All authors contributed to the article and approved the submitted version.
