# Supplementary material for: Sex-specific association of low-renin hypertension with metabolic and musculoskeletal health in Korean older adults
Source: Front Public Health. 2024 Feb 12;12:1250945. doi: 10.3389/fpubh.2024.1250945 (PMC10894919; doi:10.3389/fpubh.2024.1250945)
Supplement: Supplementary file 1 [file Table_1.DOCX]

Supplementary Material

Sex-specific association of low-renin hypertension with metabolic and musculoskeletal health in Korean older adults

**Seunghyun Lee^1^, Jae Seung Chang^2, 3^, Kyu-Sang Park^3^, Sang-Baek Koh^4^, Moon Young Kim^5, 6, 7^, Jung Soo Lim^1^**

^1^ Division of Endocrinology and Metabolism, Department of Internal Medicine, Yonsei University Wonju College of Medicine, Wonju, Republic of Korea

^2^ Department of Sports Science, Hannam University, Daejeon, Republic of Korea

^3^ Department of Physiology, Yonsei University Wonju College of Medicine, Wonju, Republic of Korea

^4^ Department of Preventive Medicine, Yonsei University Wonju College of Medicine, Wonju, Republic of Korea

^5^ Division of Gastroenterology and Hepatology, Department of Internal Medicine, Yonsei University Wonju College of Medicine, Wonju, Republic of Korea

^6^ Regeneration Medicine Research Center, Yonsei University Wonju College of Medicine, Wonju, Republic of Korea

^7^ Cell Therapy and Tissue Engineering Center, Yonsei University Wonju College of Medicine, Wonju, Republic of Korea

*** Correspondence:**Jung Soo Lim

Division of Endocrinology and Metabolism, Department of Internal Medicine, Institute of Evidence-based Medicine, Yonsei University Wonju College of Medicine, Wonju, Korea

20, Ilsan-ro, Wonju-si, Gangwon-do, 26426, Republic of Korea

Email: isiss21@yonsei.ac.kr

Tel.: +82-33-741-0530

ORCID: <https://orcid.org/0000-0003-4856-3462>

**Supplementary Table 1.** **Baseline characteristics analyzed by gender.**

|  | Men (n=86) | | | Women (n=170) | | |
| --- | --- | --- | --- | --- | --- | --- |
|  | LRH (n=22) | Non-LRH (n=64) | P-value | LRH (n=67) | Non-LRH (n=103) | P-value |
| Age (y) | 67 [65–74] | 70 [64–75] | 0.890 | 66 [61–72] | 64 [60–69] | 0.070 |
| HTN med (n, %) | 10 (45.5%) | 33 (51.6%) | 0.621 | 42 (62.7%) | 34 (33.0%) | <0.001* |
| DM (n, %) | 4 (18.2%) | 24 (37.5%) | 0.095 | 6 (9.0%) | 18 (17.5%) | 0.119 |
| Dyslipidemia (n, %) | 13 (59.1%) | 41 (64.1%) | 0.677 | 49 (73.1%) | 56 (54.4%) | 0.014* |
| Osteoporosis (n, %) | 0 (0.0%) | 3 (4.7%) | 0.301 | 24 (35.8%) | 27 (26.2%) | 0.182 |
| PRA (ng/mL) | 0.56 [0.34–0.75] | 1.61 [0.91–6.35] | <0.001* | 0.59 [0.32–0.74] | 1.01 [0.53–1.94] | <0.001* |
| Aldosterone (pg/mL) | 14.1 [11.2–19.0] | 14.4 [11.8–19.4] | 0.774 | 16.1 [13.0–19.1] | 15.2 [11.9–18.7] | 0.255 |
| Angiotensin II (pg/mL) | 45.0 [34.4–79.3] | 73.7 [54.6–98.6] | 0.020* | 73.0 [53.7–123.0] | 78.6 [58.9–115.5] | 0.599 |
| AST (U/L) | 24 [20–27] | 24 [20–28] | 0.976 | 22 [19–25] | 22 [19–25] | 0.540 |
| ALT (U/L) | 20 [16–23] | 20 [16–27] | 0.555 | 18 [13–22] | 17 [13–21] | 0.235 |
| Cr (mg/dL) | 1.0 [0.8–1.0] | 0.9 [0.8–1.0] | 0.649 | 0.7 [0.6–0.8] | 0.7 [0.6–0.7] | 0.412 |
| *Metabolic stress-related biomarkers* | | | | | | |
| RBP4 (µg/mL) | 29.8 [23.0–36.6] | 32.9 [28.0–42.2] | 0.235 | 29.3 [26.0–35.3] | 29.7 [24.5–33.8] | 0.643 |
| IL6 (pg/mL) | 1.55 [1.25–2.61] | 1.47 [1.05–2.50] | 0.546 | 1.69 [1.11–2.41] | 1.42 [1.00–2.22] | 0.108 |
| Mstn | 2.79 [2.33–3.62] | 2.89 [2.31–3.26] | 0.635 | 2.65 [1.98–3.16] | 2.37 [1.84–3.10] | 0.419 |
| Tgfb1 | 25.3 [22.6–31.2] | 24.3 [20.3–29.6] | 0.520 | 23.6 [19.2–27.5] | 24.3 [19.2–30.4] | 0.510 |
| DCN | 7.61 [6.51–8.36] | 7.19 [6.31–7.85] | 0.303 | 6.92 [5.89–8.16] | 6.75 [5.83–7.79] | 0.399 |
| GDF15 (pg/mL) | 932 [807–1054] | 1116 [747–1408] | 0.322 | 803 [690–1027] | 774 [618–1086] | 0.471 |
| FGF19 (pg/mL) | 161 [120–259] | 159 [115–382] | 0.988 | 171 [101–312] | 174 [109–312] | 0.953 |
| FGF21 (pg/mL) | 128 [95–196] | 181 [111–312] | 0.107 | 262 [165–355] | 185 [124–301] | 0.013* |
| *Metabolic parameters* | | | | | | |
| BMI (kg/m^2^) | 24.4 [23.3–26.2] | 25.0 [23.7–27.1] | 0.507 | 24.4 [23.2–26.4] | 24.3 [22.7–26.2] | 0.725 |
| Total fat (%) | 28.4 [27.4–31.8] | 29.1 [27.5–31.5] | 0.607 | 40.9 [37.0–42.0] | 40.0 [37.1–42.0] | 0.720 |
| Glucose (mg/dL) | 103 [96–117] | 104 [94–115] | 0.905 | 97 [91–103] | 97 [93–105] | 0.407 |
| Triglyceride (mg/dL) | 127 [93–166] | 152 [109–220] | 0.200 | 112 [86–177] | 118 [89–176] | 0.597 |
| Total cholesterol (mg/dL) | 173±40 | 165±38 | 0.387 | 181±33 | 183±32 | 0.712 |
| HDL cholesterol (mg/dL) | 49.5 [38.0–56.0] | 43.5 [39.5–54.0] | 0.411 | 54 [49–63] | 54 [45–65] | 0.758 |
| C-peptide (ng/mL) | 2.2 [1.9–4.4] | 2.6 [1.9–3.4] | 0.729 | 1.9 [1.4–2.6] | 2.1 [1.4–2.9] | 0.300 |
| Insulin (uIU/mL) | 8.1 [4.4–20.7] | 8.1 [5.6–13.1] | 0.921 | 6.4 [4.7–11.2] | 6.7 [5.0–10.5] | 0.508 |
| HOMA-IR | 2.1 [1.1–5.4] | 2.2 [1.5–4.0] | 0.968 | 1.5 [1.0–2.6] | 1.6 [1.1–2.7] | 0.367 |
| HOMA-β | 76 [46–136] | 68 [41–108] | 0.452 | 73 [52–109] | 69 [50–105] | 0.881 |
| Adiponectin | 5.9 [2.5–7.7] | 4.2 [2.9–7.1] | 0.621 | 8.4 [4.3–12.8] | 7.1 [4.3–11.0] | 0.279 |
| Leptin | 3.4 [2.3–5.9] | 3.8 [2.5–5.5] | 0.729 | 11.2 [7.8–16.1] | 10.4 [6.3–15.4] | 0.430 |
| *Musculoskeletal parameters* | | | | | | |
| Ca (mg/dL) | 9.5±0.3 | 9.5±0.3 | 0.310 | 9.5±0.3 | 9.6±0.4 | 0.124 |
| P (mg/dL) | 3.5±0.4 | 3.6±0.4 | 0.566 | 3.8±0.4 | 3.9±0.4 | 0.323 |
| ALP (IU/L) | 65 [54–75] | 67 [56–82] | 0.170 | 67 [59–82] | 71 [57–81] | 0.717 |
| ALM index (kg/m^2^) | 6.98 [6.61–7.45] | 6.88 [6.56–7.52] | 0.832 | 5.41 [5.10–5.82] | 5.35 [5.00–5.75] | 0.845 |
| Handgrip strength (kg) | 38.8 [34.2–42.5] | 38.3 [33.3–42.8] | 0.851 | 21.7 [18.8–24.8] | 23.5 [19.5–26.6] | 0.069 |
| Gait speed (m/sec) | 1.08 [0.97–1.20] | 1.07 [0.98–1.18] | 0.968 | 1.03 [0.92–1.16] | 1.04 [0.93–1.14] | 0.832 |
| Chair stand test (sec) | 10.0 [7.9–12.5] | 9.4 [7.5–10.7] | 0.293 | 11.5 [8.8–13.6] | 10.0 [8.3–12.5] | 0.167 |
| TUG time (sec) | 9.5 [8.3–10.8] | 8.8 [7.8–9.5] | 0.077 | 8.7 [8.2–10.5] | 8.7 [7.9–10.1] | 0.314 |
| SPPB (score) | 12 [11–12] | 12 [12–12] | 0.134 | 11 [10–12] | 12 [11–12] | 0.031* |
| TBS | 1.42 [1.39–1.45] | 1.41 [1.36-1.46] | 0.523 | 1.31 [1.27–1.35] | 1.33 [1.29–1.38] | 0.037* |
| Lumbar spine T-score | 0.2 [-0.4 to 1.3] | -0.2 [-1.0 to 0.7] | 0.104 | -1.5 [-2.4 to -0.7] | -1.5 [-2.2 to -0.7] | 0.880 |
| Femur neck T-score | -0.2 [-1.0 to 0.2] | -0.8 [-1.4 to 0.1] | 0.296 | -1.9 [-2.4 to -1.4] | -1.5 [-2.2 to -1.0] | 0.019* |
| Total hip T-score | 0.2 [-0.7 to 0.8] | 0.1 [-0.7 to 0.5] | 0.513 | -0.9 [-1.5 to -0.5] | -0.9 [-1.3 to -0.2] | 0.211 |
| Fracture history (n, %) | 6 (27.3%) | 15 (24.6%) | 0.804 | 12 (18.5%) | 12 (12.1%) | 0.261 |
| Nontraumatic fracture history (n, %) | 1 (4.6%) | 4 (6.6%) | 0.734 | 6 (9.2%) | 6 (6.0%) | 0.435 |

* indicates a significant difference p<0.05. Abbreviations: LRH, low renin hypertension; y, years; BMI, body mass index; HTN, hypertension; DM, diabetes mellitus; HOMA-IR, Homeostatic Model Assessment for Insulin Resistance; HOMA-β, Homeostatic Model Assessment for beta cell function; Tg, triglyceride; AST, aspartate aminotransferase; ALT, alanine aminotransferase; Ca, calcium; P, phosphorus; Cr, creatinine; TBS, trabecular bone score.
